# The potential value of cancer‐testis antigens in ovarian cancer: Prognostic markers and targets for immunotherapy

**DOI:** 10.1002/iid3.1284

**Published:** 2024-06-19

**Authors:** Lina Lin, Xiaoqiong Zou, Weixia Nong, Yingying Ge, Feng Li, Bin Luo, Qingmei Zhang, Xiaoxun Xie

**Affiliations:** ^1^ Department of Histology and Embryology, School of Basic Medicine Science Guangxi Medical University Nanning Guangxi People's Republic of China; ^2^ Department of Obstetrics and Gynecology The First Affiliated Hospital of Guangxi Medical University Nanning Guangxi People's Republic of China; ^3^ Education Department of Guangxi Zhuang Autonomous Region Key Laboratory of Basic Research on Regional Diseases (Guangxi Medical University) Nanning Guangxi People's Republic of China; ^4^ Ministry of Education, Key Laboratory of Early Prevention and Treatment of Regional High Frequency Tumor (Guangxi Medical University) Nanning Guangxi People's Republic of China

**Keywords:** CTA, ovarian cancer, tumor therapy

## Abstract

**Background:**

Tumor immunotherapy has become an important adjuvant therapy after surgery, radiotherapy, and chemotherapy. In recent years, the role of tumor‐associated antigen (TAA) in tumor immunotherapy has become increasingly prominent. Cancer‐testis antigen (CTA) is a kind of TAA that is highly restricted in a variety of tumors and can induce an immune response.

**Aims:**

This review article aimed to evaluate the role of CTA on the progression of ovarian cancer, its diagnostic efficacy, and the potential for immunotherapy.

**Methods:**

We analyzed publications and outlined a comprehensive of overview the regulatory mechanism, immunogenicity, clinical expression significance, tumorigenesis, and application prospects of CTA in ovarian cancer, with a particular focus on recent progress in CTA‐based immunotherapy.

**Results:**

The expression of CTA affects the occurrence, development, and prognosis of ovarian cancer and is closely related to tumor immunity.

**Conclusion:**

CTA can be used as a biomarker for the diagnosis and prognosis evaluation of ovarian cancer and is an ideal target for antitumor immunotherapy. These findings provide novel insights on CTA in the improvement of diagnosis and treatment for ovarian cancer. The successes, current challenges and future prospects were also discussed to portray its significant potential.

## Graphical abstract

Cancer‐testis antigens (CTAs) exhibit carcinogenic potential and strong immunogenicity in ovarian cancer. This review outlines a comprehensive of overview the regulatory mechanism, immunogenicity, clinical expression significance, tumorigenesis, and application prospects of CTAs in ovarian cancer, all of which can provide novel insights in the improvement of diagnosis and treatment for ovarian cancer.

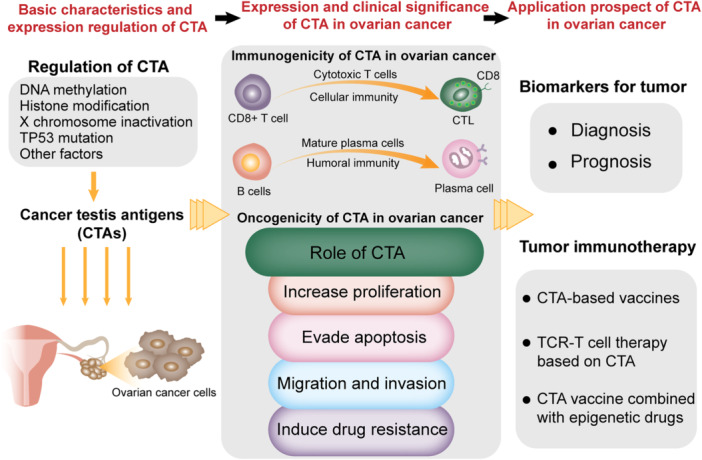

## 1 INTRODUCTION

Ovarian cancer is the deadliest gynecological malignancy, the second most common malignancy in women over the age of 40, and the fifth leading cause of cancer death in women.^1^ According to the latest statistics from the National Cancer Center, ovarian cancer accounts for approximately 2.93% of new cases and 2.24% of deaths in China each year.^2^ In the past decade, the mortality of ovarian cancer in China has shown an upward trend mainly because of its hidden onset and lack of accurate and effective early screening methods. More than 70% of patients are in the advanced stage of the disease when they are diagnosed.^3^, ^4^ The treatment of advanced ovarian cancer is limited and has a poor effect, which is often difficult to cure. Recurrence and metastasis occur in a short time, seriously affecting the quality of life of patients, and the 5‐year survival rate of patients is only 30%. Thus, early diagnosis and maintenance therapy after surgical resection are particularly important in ovarian cancer, and novel treatment methods are urgently needed to improve the prognoses of patients with advanced ovarian cancer.^5^ Hence, current research has focused on the therapeutic targets and related molecular mechanisms of ovarian cancer. Owing to the development of whole genome sequencing technology, the genome map of ovarian cancer has been established, which facilitates the identification of the driving genes of ovarian cancer occurrence and development and provides a strategy for the targeted treatment of ovarian cancer.^6^

**Figure 1:**
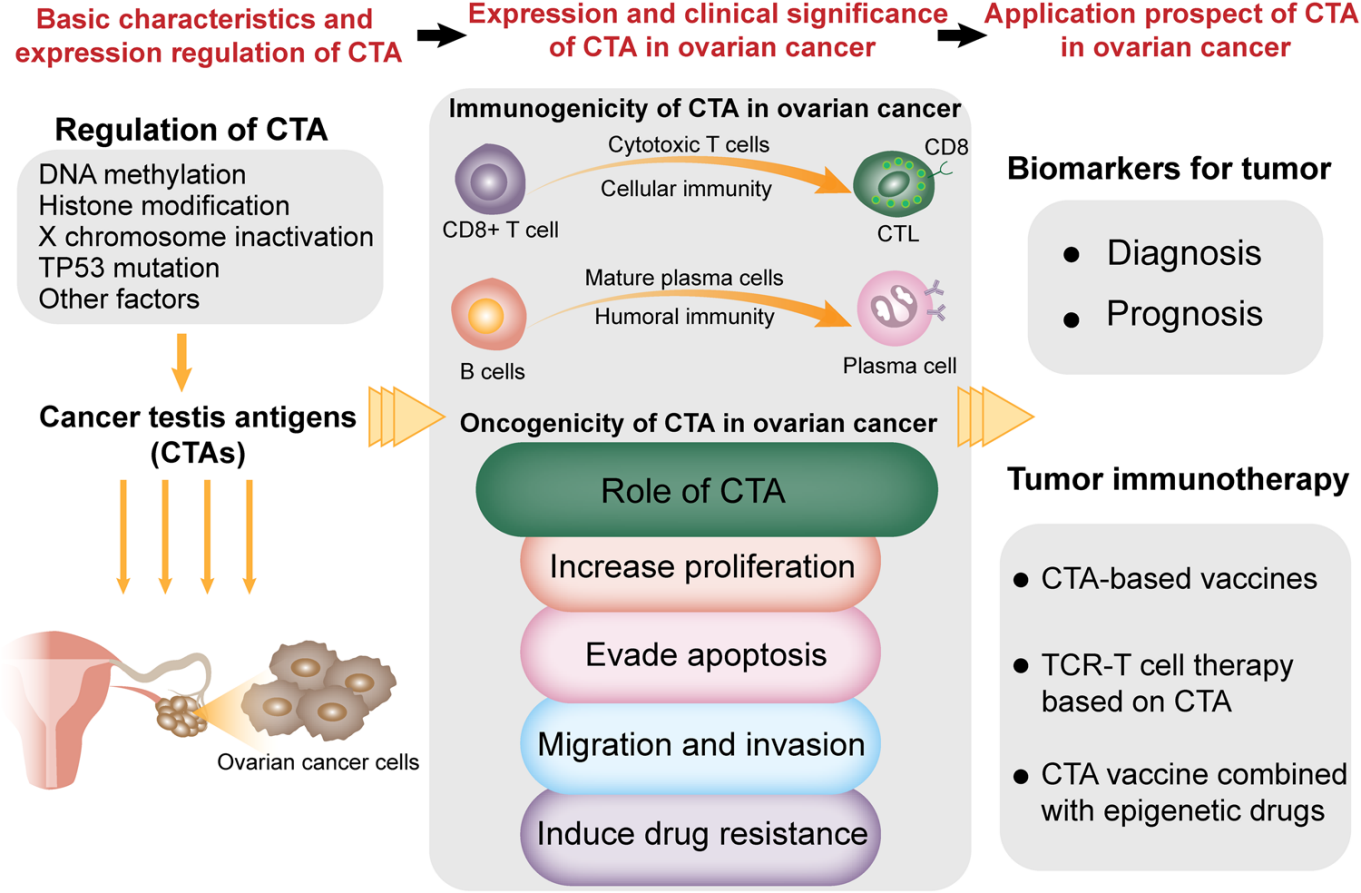
Schematic illustration of the oncogenic functions versus immunogenic of cancer‐testis antigens and their potential clinical applications in ovarian cancer. CTAs exhibit carcinogenic potential in ovarian cancer. They are possibly involved in steps of tumorigenesis that induction of proliferation, evasion of apoptosis, enhancement of tumor migration (invasion and metastasis), and drug resistance. At the same time, CTAs that are expressed on the surface of ovarian cancer cells may have immunogenic and oncogenic properties. The strongly immunogenic CTAs could induce an expansion of CD8^+^ T cells that are capable of eliciting cellular immunity or activate B cells that induce a humoral response in the form of tumor‐specific antibodies. CTAs, cancer‐testis antigens; CTL, cytotoxic T lymphocyte; TCR‐T, T cell receptor‐engineered T.

Cancer‐testis antigen (CTA) is a kind of tumor‐associated antigen (TAA). The CTA gene belongs to a family of genes abnormally expressed in embryonic stem cells and a variety of malignant tumors but are not expressed or restrictedly expressed in somatic cells and normal tissues (except the testis).^7^ CTA has attracted substantial interest because of its unique expression pattern and high immunogenicity in tumors. It not only can be used as a biomarker for tumor diagnosis and prognosis evaluation but also is considered an ideal target for tumor immunotherapy. CTA is possibly involved in many events in ovarian cancer, such as the generation of tumor stem cell‐like cells, participation of epithelial–mesenchymal transition (EMT), and enhancement of the invasion and metastasis ability of tumor cells, all of which may play an important role in the initiation and maintenance of tumor development.^8^ This review will focus on recent progress in research into CTA in ovarian cancer to provide a theoretical basis for exploring a CTA‐based treatment strategy for ovarian cancer (Figure 1).

## 2 BASIC CHARACTERISTICS AND EXPRESSION REGULATION OF CTA

To date, 276 CTA genes or gene families have been documented by the CTdabase (http://www.cta.lncc.br). In general, CTA can be divided into two categories: CT‐X antigens that are encoded by linked genes located on the X chromosome and non‐X CT antigens encoded by genes located on the autosome or Y chromosome.^9^ In a normal testis, the CT‐X gene is usually expressed in the spermatogonium stage (early stage) of spermatogenesis and contributes to sperm development from mitosis to maturity. The non‐X CT gene is usually expressed in the spermatocyte stage (late stage) of spermatogenesis and is involved in male germ cell differentiation. Unlike non‐X CT antigens, CT‐X antigens form closely related gene family clusters that are activated frequently in tumor cells, and their expression is often associated with advanced tumors with poor prognoses. In summary, the CTA gene has the following common characteristics: (1) restricted expression, that is, high expression in a variety of malignant tumors but not in normal tissues (except the testis, fetal ovary, and placental germ cells); (2) expression regulated commonly by epigenetic, such as DNA methylation; and (3) immunogenicity.

Given that the expression characteristics of CTA are considered ideal targets for tumor immunotherapy, understanding its expression regulation mechanism will benefit its application to tumor immunotherapy. In general, the regulation mechanism of CTA involves two aspects: epigenetic factors and nonepigenetic factors. A large number of studies have shown that epigenetic regulation is the key mechanism of CTA gene transcription regulation, mainly including DNA methylation and histone modification.^10^

### 2.1 DNA methylation

DNA methylation is one of the main mechanisms of gene silencing and DNA demethylation is often associated with the reactivation of silenced genes. Changes in methylation status can lead to disturbances in genomic homeostasis, leading to diseases and tumor development. The methylation of CpG island in the promoter regions of many CTA genes is crucial to the expression regulation of CTA genes, and thus DNA demethylation plays a leading role in activating CTA gene expression.^11^, ^12^ In addition, the expression level of a CTA gene is correlated with change in genome‐wide DNA methylation status in tumors.^13^ Multiple CT‐X antigens (e.g., MAGEA, CT1; GAGE, CT4; NY‐ESO‐1, CT6; PAGE, CT16; and TSPY1, CT78) are highly expressed in prostate cancer accompanied by DNA demethylation, and changes in DNA methylation status are associated with tumor metastasis.^14^ The expression of the HAGE gene in myeloid leukemia is related to the methylation status of its promoter.^15^ The demethylation of the CAGE (CT95) gene promoter leads to its abnormal expression in gastric cancer.^16^ Woloszynska‐Read et al.^17^, ^18^ reported that DNA demethylation drives BORIS (CT27) expression in ovarian cancer. Similarly, the expression of NY‐ESO‐1(CT6), MAGE, and other CTA genes in ovarian cancer is closely related to promoter methylation and overall DNA methylation.^19^, ^20^ A study showed the association between CT45 promoter demethylation and its expression level in epithelial ovarian cancer, reporting a significant decrease in CT45 promoter methylation in CT45 protein–positive tumors and DNA methylation inhibited the promoter activity of CT45 gene. Furthermore, demethylation by DNA methyltransferase inhibitor (5‐aza‐2′‐deoxycytiside, DAC) can induce increase in CT45 mRNA and protein expression in ovarian cancer cells. DAC treatment can enhance the sensitivity of ovarian cancer cells to platinum drugs.^21^ Moreover, Zhang et al.^22^ identified PRAME exhibits frequent expression in epithelial ovarian cancer (EOC) at both the mRNA and protein levels, with DNA methylation serving as a critical mechanism in the regulation of its expression. DNA methylation also activates CTA genes by regulating nucleosome occupation. In conclusion, the expression of CTA gene in ovarian cancer largely depends on abnormal DNA demethylation.

### 2.2 Histone modification

The posttranscriptional modification of histones is one of the important epigenetic regulatory mechanisms. It plays a key role in controlling the expression of tumor genes, along with DNA methylation. The inhibited expression of many CTAs (such as NY‐ESO‐1, MAGE‐A1, and MAGE‐A3) in tumor cells is consistent with DNA hypermethylation and histone heterochromatin modification in the promoters of these genes. In addition, the demethylation and re‐expression of CTA genes are usually associated with the loss of inhibitory histones and increase in active histones. The inhibition of histone lysine deacetylation can enhance the activation and expression of a CT‐X gene. Under the action of methyltransferase and deacetylase (HDAC), histones are methylated and acetylated and bind to methyl‐CpG proteins to directly activate CTA genes.^23^, ^24^ This process has been observed in ovarian cancer. Steele et al.^25^ found that the expression of MAGE‐A1 significantly increased in vitro and in vivo after the ovarian cancer platinum‐resistant cell line A2780/cp70 treated with DAC and HDAC inhibitors. DAC combined with HDAC inhibitors significantly enhanced the chemosensitivity of ovarian cancer to platinum. Similarly, DNA demethylation and histone acetylation promote the expression of the multiple subtypes of the BORIS family in ovarian cancer cells,^18^ indicating that histone modification and DNA methylation can synergically regulate the expression of CTA genes in ovarian cancer.

### 2.3 TP53 mutation

TP53 mutations are common in high‐grade serous ovarian cancer. PLAC1 is also known as CT92. A study has shown that wild‐type TP53 as a transcriptional factor can inhibit PLAC1 transcription, whereas mutant or deleted TP53 cannot recognize the binding site of the P1 promoter of PLAC1 and thus lose the transcriptional inhibition of PLAC1 transcription. Therefore, TP53 status affects PLAC1 expression in serous ovarian cancer.^26^ Another study showed that wild‐type TP53 negatively regulated BORIS expression by binding BORIS promoter and interacting with the transcription factor Sp1 in epithelial ovarian cancer; this relationship may be the reason for the high expression of BORIS in epithelial ovarian cancer.^27^ Overall, TP53 mutation influences the activation and expression of CTA genes.

### 2.4 X chromosome inactivation (XCI)

XCI is the epigenetic inactivation of one of two X chromosomes in XX eutherian mammals. The loss of XCI is thought to have an oncogenic effect in human malignancies, including breast and ovarian cancer, and XCI is associated with CTA expression in ovarian cancer.^28^, ^29^ Kang et al.^30^ found that the CT‐X antigens MAGEA4 and XAGE3 were upregulated by loss of XCI in high‐grade serous ovarian cancer and may have a role in tumor aggressiveness. To date, only this study reported the XCI‐induced aberrant expression of CTA in ovarian cancer, and the specific mechanism needs to be further studied.

### 2.5 Other factors

Cytokines, tyrosine kinases, and specific transcription factors are possibly involved in the regulation of CTA expression. For example, IL‐7 and GM‐CSF can upregulate SPAN‐xb expression in multiple myeloma, and the tyrosine protein kinase KIT induces the transcription of the MAGE gene in ovarian cancer.^31^, ^32^

## 3 EXPRESSION AND CLINICAL SIGNIFICANCE OF CTA IN OVARIAN CANCER

In recent years, many studies have focused on the expression and clinical significance of CTA in ovarian cancer. The members of the CTA family in ovarian cancer mainly include MAGE, GAGE 1/2, LAGE‐1, NY‐ESO‐1, SSX, CT45, and TRAG‐3, which belong to the CT‐X antigen, and AKAP3/4, BAGE, BORIS, OY‐TES‐1(CT23), PRAME, PIWIL, HSP70‐2, SPAG9, and SP17, which belong to non‐X CT antigen.^8^ The characteristics of CTAs are summarized in Table 1.

**Table 1:** Characteristics of cancer‐testis antigens (CTAs) in ovarian cancer (OC).

CTA genes	Aliases	Location	Clinical specimens/cell lines	Expression frequency in OC (%)	Immune response in OC	CTA oncogenic function	Prognosis	References
ACRBP	CT23, OY‐TES‐1sp32	12p12‐13	OC tissuesOC SerumSKOV3,A2780	mRNA 32.3% (20/62) mRNA or protein 69% (69/100) Protein 81% (87/107) Protein 10%(1/10), 28.5% (16/56)	Humoral and cellular immune response	Migration and invasionCell proliferation, karyomitosis, paclitaxel resistance	OS, PFS, FIGO stage	[33, 34, 35, 36, 37]
AKAP3	CT82	12p13.3	OC tissuesSKOV3OVCA432	mRNA 58% (43/74), 28% (15/54)	Unknown	Migration and invasion	OS, PFS	[38, 39]
AKAP4	CT99	Xp11.2	OC tissues	mRNA or protein 89% (34/38)	Humoral immune response	DNA damage, cell cycle, apoptosis, EMT	Unknown	[40]
BAGE	CT2	21p11.2	OC ascites specimensOC tissues	mRNA 63% (17/27) mRNA 14.6% (6/41), 19.4% (12/62)	Cellular immune response	Unknown	Positive in OC patients with ascites	[35, 41, 42, 43]
BORIS	CT27, CTCFL	20q13.31	OC tissuesOVCAR8	mRNA 82.6% (62/75)	Unknown	DNA hypomethylation, tumor progression	OS, PFS, FIGO stage	[17, 18, 20, 27]
CT45	CT45	Xq26.3	OC tissuesOVCAR5	mRNA 25% (29/118) Protein 37% (82/219)	Unknown	DNA damage repair, platinum sensitivity	OS, PFS, FIGO stage	[21, 35, 44, 45]
GAGE‐1/2	CT4	Xp11.23	OC ascites specimensOC tissuesA2780	mRNA 30% (8/27), mRNA 26.8% (11/41), Protein 14.8% (46/310)	Unknown	Paclitaxel resistant	Positive in OC patients with ascites	[35, 41, 42, 43, 46]
HOM‐TES‐85	CT28, LUZP4	Xq23	OC tissues	mRNA 32% (7/22)	Humoral immune response	Transcriptional activity	Unknown	[47]
HORMAD1	CT46	1q21.2	OC tissuesSKOV3,A2780ES‐2	mRNA 76.1% (68/90)	Unknown	Migration and invasion cell cycle regulation, apoptosis, angiogenesis	Unknown	[48]
HSP70‐2	HSPA2	6p21.33	A‐10, Caov‐3, SKOV3	Unknown	Unknown	Cell cycle, apoptosis, EMT	Unknown	[49, 50]
LAGE‐1	CT6CTAG2	Xq28	OC tissuesSKOV3	mRNA 21% (22/107), 25.8% (16/62)	Humoral immune response	Unknown	FIGO stage	[35, 51]
MAGE‐A1	CT1.1	Xq28	OC ascites specimensOC tissuesES‐2, OVK‐18	mRNA 7% (2/27) mRNA 55% (15/27), 40.3% (25/62), mRNA or protein 15% (42/281) Protein 60% (3/5)	Humoral and cellular immune response	Migration and invasion, cell proliferation	PFS positive in OC patients with ascites	[35, 41, 42, 43, 46, 52, 53]
MAGE‐A2	CT1.2	Xq28	OC tissues	mRNA 9% (5/58)	Unknown	Cell proliferation, paclitaxel resistance	Unknown	[46, 52]
MAGE‐A3	CT1.3	Xq28	OC ascites specimensOC tissuesA2780, COC1	mRNA 30% (8/27) mRNA 40.3% (25/62), mRNA or protein 36% (131/390)	Cellular immune response	Cell proliferation, paclitaxel resistance	FIGO stage, positive in OC patients with ascites	[35, 41, 42, 52, 53, 54, 55, 56, 57]
MAGE‐A4	CT1.4	Xq28	OC tissuesOC SerumFraW, KlHe	mRNA 64.5% (40/62), 16% (3/19), mRNA or protein 47% (186/399), 34.2% (200/585) Protein 22% (13/60)	Humoral immune response	Coregulation with other MAGE genes	OS, PFS	[35, 52, 53, 58]
MAGE‐A9	CT1.9	Xq28	OC tissues	Protein 36.7% (47/128)	Unknown	Unknown	OS, grade, FIGO stage, CA‐125, metastasis	[59]
MAGE‐A10	CT1.10	Xq28	OC tissues	mRNA 11.3% (7/62) mRNA or protein 52% (204/395)	Unknown	Coregulation with other MAGE genes	OS, PFS	[35, 53]
MAGE‐C1	CT7.1	Xq27	OC tissues	mRNA or protein 16% (42/267) protein 35% (28/80), 24% (56/230)	Unknown	Coregulation with other MAGE genes	OS, PFS	[35, 53]
NY‐ESO‐1	CT6	Xq28	OC tissuesSKOV3, 2780, OVCAR3	mRNA 25.9% (260/1002) 30% (32/107), 8.1% (5/62) Protein 43% (62/143), 26.5% (232/874), 22.6%(132/585)	Humoral and cellular immune response	Coregulation with other CT genes	OS, PFS, FIGO stage, grade	[35, 51, 60, 61, 62, 63, 64, 65]
PIWIL1	CT80.1, HIWI	12q24.33	OC tissuesA2780	mRNA 61% (129/211) Protein 92% (11/12)	Unknown	Migration and invasion, angiogenesis	FIGO stage, grade, metastasis	[66, 67, 68, 69]
PIWIL2	CT80.2	8p21.3	OC tissues	mRNA 75% (3/4)	Unknown	DNA damage, platinum resistance, apoptosis	FIGO stage	[67, 70]
PRAME	CT130	22q11.22	OC tissuesOVCAR3	mRNA 60% (70/119) Protein 90% (43/48), 63% (15/24)	Unknown	Migration and invasion, DNA hypomethylation	OS, DFS, FIGO stage, metastasis	[22, 71, 72]
PLAC1	CT92	Xq26	OC tissuesEFO‐21, Caov‐3	mRNA 21% (21/101)	Unknown	Unknown	Unknown	[73, 74]
POTE	CT104.2	2q21.1	OC tissuesOVCAR3, A2780, OVCAR429	mRNA 33% (38/114)	Unknown	DNA hypomethylation	OS	[75]
SPAG9	CT89	17q21.33	OC tissuesA‐10, SKOV6, Caov‐2	mRNA 90% (18/20) Protein 90% (18/20)	Humoral immune response	Unknown	FIGO stage, metastasis	[76]
SP17	CT22, SPA17	11q24.2	OC tissuesSKOV3, ES‐2, HO8910	mRNA 43.5% (27/62) Protein 43% (30/70)	Humoral and cellular immune response	Migration and invasion, chemosensitivity	OS	[35, 77, 78, 79]
SSX2	CT5	Xp11.22	OC tissuesSVOV3, OVCAR3	mRNA 10% (12/122)	Humoral immune response	Unknown	Unknown	[35, 80, 81]
TAG	CT49	5p15.2	OC tissuesSVOV3, ES‐2, OVCAR3, Caov‐3, OV90	mRNA 35% (8/23)	Cellular immune response	Unknown	Unknown	[82]
TRAG‐3	CT24, CSAG2	Xq28	OC tissuesOVCAR4	Protein 84% (31/37)	Unknown	Chemosensitivity	OS, PFS	[83, 84]
TEX19	FLJ35767	17q25.3	OC tissuesOVCAR3, A2780, HO8910	Unknown	Cellular immune response	Cell proliferation, migration, and invasion	FIGO stage, depth of invasion, lymph node metastasis	[85]
XAGE1	CT12GAGED2	Xp11.22	OC tissuesOVCAR	mRNA 4.8% (3/62)	Unknown	DNA hypomethylation	OS,FIGO stage, histological type	[35, 86]

*Note*: Ovarian cancer cell lines: SKOV3, A2780, OVCA432, OVCAR8, OVCAR5, ES‐2, OVK‐18, COC1, FraW, KlHe, OVCAR3, EFO‐21, Caov‐3, OVCAR429, A‐10, SKOV6, Caov‐2, OVCAR4, OVCAR, SVOV3, OV90.

Abbreviations: FIGO, 2018 International Federation of Gynecology and Obstetrics; mRNA, messenger RNA; OS, overall survival; PFS, progression‐free survival.

Among CTAs, the MAGE genes represent the largest and most classic gene family. MAGE has attracted increasing attention from researchers. During the past several years, many researchers have studied the expression of MAGE genes in ovarian cancer.^52^ In a study on the role of MAGE family in ovarian cancer, the researchers examined multiple MAGE family antigen genes (MAGE‐A1, MAGE‐A3, MAGE‐A4, MAGE‐A10, and MAGE‐C1) and found that 78% of epithelial ovarian cancer expressed at least one MAGE antigen. The expression frequencies of each antigen were as follows: MAGE‐A1, 15% (42/281); MAGE‐A3, 36% (131/390); MAGE‐A4, 47% (186/399); MAGE‐A10, 52% (204/395); and MAGE‐C1, 16% (42/267). The expression level of MAGE‐A1, MAGE‐A3, MAGE‐A4, and MAGE‐A10 were correlated with poor prognosis, whereas the expression level of MAGE‐C1 was not.^53^ Similar results were confirmed by Zhang et al.,^41^ MAGE‐1 and MAGE‐3 had high expression rates of 53.7% and 36.6%, while GAGE‐1/2 and BAGE had lower rates of 26.8% and 14.6% in ovarian cancer tissues. Other studies showed that the positive rate of MAGE‐A4 protein in serum was 22% in primary ovarian cancer.^58^ Similarly, the expression levels of MAGE‐A9 mRNA and protein were considerably elevated in ovarian cancer and were strongly associated with FIGO stage, tumor metastasis, and poor prognosis.^59^ In addition, the MAGE‐A3/6 protein was found in the plasma exosomes of patients with ovarian cancer, and the expression rate of MAGE‐A3 in the ascites of the patients was 30%.^54^ Coincidentally, Hofmann et al.^42^ found that testing for BAGE, GAGE‐1/2, MAGE‐1, and MAGE‐3 mRNAs in ascites specimens of ovarian cancer patients results in high sensitivity in diagnosing malignant ascites. Szender et al.^60^ showed that 40.7% of 1002 ovarian cancer cases express NY‐ESO‐1 mRNA or protein. Patients with NY‐ESO‐1‐positive tumor tend to be older, at the late stage (85% III/IV), with high grade (G3), more serous histotype, and poor response to initial treatment. These patients tended to have short overall survival and progression‐free survival, suggesting association between NY‐ESO‐1 expression and an aggressive cancer phenotype. Odunsi et al.^51^ found that the expression of NY‐ESO‐1 or LAGE‐1 in epithelial ovarian cancer was approximately 50%, and the coexpression of both accounted for 11%. CT45 is not expressed in normal ovarian and nonepithelial cancer types but significantly expressed in a significant proportion of epithelial ovarian cancer types and associated with FIGO stage, serous histological type, and tumor grade.^21^, ^44^ The POTE family belongs to the CT104 family in the CT database. POTE is significantly overexpressed in epithelial ovarian cancer and correlated with FIGO stage and tumor grade. Subgroup analysis showed that the high‐grade serous ovarian cancer had the highest expression rate of POTE.^75^ Xu et al.^85^ evaluated TEX19 expression by immunohistochemistry in 98 human ovarian tissue samples, in which TEX19 was significantly higher than that in adjacent normal tissues, and its expression level was related to tumor stage, infiltrating depth, and lymph node metastasis. Regarding OY‐TES‐1 (CT23), Tammela et al.^33^ first reported 60% of its protein expression in epithelial ovarian cancer. Subsequently, we found that the expression of OY‐TES‐1 protein in ovarian cancer was 81%, the positive rate of epithelial ovarian cancer was 66.4% (71/107), and the positive rate of nonepithelial ovarian cancer was 14.9% (16/107).^34^ Other studies showed that the expression of XAGE1 (CT12 family) in ovarian cancer was higher than that in benign ovarian tumor (least signifcant diﬀerence = 2.319) and was correlated with stage and pathological type.^86^ A report showed high frequency of SPAG9 (CT89) mRNA in serous carcinoma (88%), mucinous carcinoma (100%), and transparent carcinoma (100%), respectively. Its protein expression had a high frequency (90%).^87^ Jagadish et al.^76^ also observed increased expression of SPAG9 in multiple ovarian cancer cells. Moreover, separate studies in ovarian cancer demonstrated that the expression rates of Sp17 (CT22), SSX (CT5), AKAP3 (CT82), AKAP4 (CT99), and PLAC1 (CT92) were 43%, 26%, 58%, 89%, and 21%, respectively.^38^, ^40^, ^73^, ^77^, ^80^ A recent large‐scale study (5800 participants) demonstrated that >50% of PRAME‐positive lesions were found among ovarian clear cell carcinomas (90%), ovarian serous carcinomas (63%).^71^ In the current study, CTA was expressed with different frequencies in different pathological types of malignant ovarian tumors but still mainly expressed in epithelial ovarian cancer. Stratified studies with large samples are needed.

In addition to separate studies on different CTAs in ovarian cancer, Garcia‐Soto et al.^35^ analyzed the coexpression of 20 CTA genes in epithelial ovarian cancer; they found that the expression of CTA in epithelial ovarian cancer was heterogeneous, and the expression of CTA in tumors was not single. The MAGE‐A4, MAGE‐A1, MAGE‐A3, OY‐TES‐1, and MAGE‐C expressions were shared in 95% of ovarian cancer cases, while the expression levels of SP17, GAGE, NY‐ESO‐1, and XAGE‐1D were found to be closely associated with an increased risk of tumor progression. Collectively, although the expression frequencies of CTA genes are diverse in ovarian cancer, some of them are highly expressed and closely related to clinicopathological features. These CTA genes offer excellent prospects for the diagnosis and malignancy evaluation and immunotherapy. However, the current number of samples is extremely small. A larger number of samples are needed for research and analysis.

## 4 IMMUNOGENICITY OF CTA IN OVARIAN CANCER

The immunogenicity of CTA has been confirmed, which can activate and induce humoral and cellular immune responses in patients with a variety of tumors, including ovarian cancer. The importance of CTA immunogenicity lies in its use as a serum tumor marker for the early diagnosis and follow‐up monitoring of ovarian cancer and as an ideal target for specific immunotherapy and anticancer vaccine design.

Since the discovery of the MAGE family, the humoral immunity of patients with tumors to MAGE has been reported in many studies. Daudi et al.^53^ observed that 9% of patients with ovarian cancer had spontaneous humoral immune response to at least one MAGE‐A antigen, and patients with MAGE humoral immune response had a poor prognosis, which may be related to the fact that the majority of antibody‐positive patients were in the advanced stage. In the MAGE family, a subfamily member MAGE‐A3 is frequently expressed in a variety of tumors, including ovarian cancer, and induces humoral and cellular immune responses. The ability of MAGE‐A3 to induce immune responses and tumor remission has been evaluated in several clinical trials.^55^ In addition, MAGE‐A3‐specific antitumor cytotoxic T lymphocytes constructed by researchers can kill epithelial ovarian cancer cells and secrete interferon γ.^56^

Among CTAs, NY‐ESO‐1 seemed to be the most immunogenic one, which can significantly induce spontaneous and specific tumor humoral and cell‐mediated immune responses. NY‐ESO‐1 antibody with different frequencies can be detected in the sera of patients with ovarian cancer, breast cancer, lung cancer, and thyroid cancer. One study reported that a humoral immune response to NY‐ESO‐1/LAGE‐1 can be detected in 1/3 of patients with ovarian cancer expressing NY‐ESO‐1/LAGE‐1.^51^ Another study showed that 104 of 302 (34.4%) patients with ovarian cancer with NY‐ESO‐1 expression had serum NY‐ESO‐1 antibody.^60^ In an immunological investigation of patients with ovarian cancer, the presence of NY‐ESO‐1 autoantibodies was associated with increased tumor‐infiltrating CD8^+^, CD4^+^, and FoxP3^+^ cells. Autoantibodies may act synergistically with tumor‐infiltrating T cells.^88^ In addition to serological antibody studies, researchers attempted to use NY‐ESO‐1 in the immunotherapy of ovarian cancer. Odunsi et al.^61^ identified a NY‐ESO‐1 peptide (NY‐ESO‐1_157–170_) with HLA class I/II antigen specificity. This peptide can induce the comprehensive immune response of body fluids and T‐cells in patients with ovarian cancer.

OY‐TES‐1 was discovered in 2001. Since then, antibodies against OY‐TES‐1 have been discovered in the sera of patients with various malignant tumors but exclusive of ovarian cancer.^89^ Garcia‐Soto et al.^35^ used OY‐TES‐1 recombinant protein to detect its antibody and found that 10% of patients with ovarian cancer had OY‐TES‐1‐specific antibodies in their peripheral blood. Recently, our research group showed that the positive rate of OY‐TES‐1 antibody in patients with ovarian cancer was 28.5%.^90^ OY‐TES‐1 is involved in the activation of cellular immune response. Although the cellular immune response of OY‐TES‐1 in ovarian cancer has not yet been reported, liver cancer and lung cancer OY‐TES‐1 can induce specific cytotoxic T lymphocytes (CTLs) that led to specific tumor lethality.^36^, ^37^, ^91^

In addition, SPAG9 is an immunogenic CTA in ovarian cancer patients. SPAG9 is located at 17q21 of human chromosomes, which is involved in the amplification and expression of tumor‐related genes and is closely related to chromosome aberrations in various cancers. Antibodies to SPAG9 can be detected in 67% of serum samples of ovarian cancer. Approximately 62.5% of patients with early stage (I–II) and 68% of patients with advanced stage (III–IV) of ovarian cancer had a strong humoral immune response to SPAG9.^87^ It has certain significance for early screening of ovarian cancer.

## 5 ROLE OF CTA IN OVARIAN CANCER

Although the role of many CTAs in tumor processes is still unclear, they appear to play a role in several important processes, such as transcriptional regulation, signal transduction, cell proliferation, and invasion. Some CTAs function as proto‐oncogenes and are involved in the maintenance of the undifferentiated state of stem cells.^92^ In recent years, in‐depth research has found that in addition to being immune targets, some CTA genes can be carcinogenic drivers, and they can be selectively expressed during tumor evolution and are independent of the immune system. These CTA genes can lead to genomic instability by causing DNA damage, impeding DNA repair, and especially inhibiting proteins associated with homologous recombination (HR). HR defects, such as the classic BRCA1/2 gene mutation, are associated with an increased risk of breast and ovarian cancer. Genomic analysis showed that these defects exist in nearly half of high‐grade serous ovarian cancer. Whether the activation of CTA genes is related to the occurrence and development of ovarian cancer should be further explored. Notably, through protein structure analysis, researchers have found that most CTAs, especially CT‐X antigens, are intrinsically disordered proteins (IDPs). Although IDPs lack stable three‐dimensional (3D) structures, they can bind to different proteins under different conditions and induce folding of different 3D structures. Therefore, IDPs play an important role in biomolecular recognition, transcriptional regulation, gene expression, cell cycle regulation, cell differentiation, and signal transduction by regulating protein networks and are often overexpressed in pathological conditions, such as cancer.^93^ These findings provide a perspective for exploring the function of CTA. According to the available research, CTA plays a carcinogenic role in the occurrence and progression of ovarian cancer through the following aspects.

### 5.1 CTA and tumor cell proliferation and apoptosis

Hsp70‐2 is a member of the HSP70 family of proteins. Zhu et al.^49^ found that HSP70‐2 is a key factor in the formation of active CDC2/Cyclin B complex during meiosis in spermatogenesis. A recent study showed that HSP70‐2 knockout leads to cell cycle arrest, senescence, and apoptosis in epithelial ovarian cancer cells and inhibited cell proliferation and colony formation. HSP70‐2‐deficient ovarian cancer cells showed the downregulation of antiapoptotic proteins and increased expression of proapoptotic genes. HSP70‐2 promotes the malignant phenotype of ovarian cancer cells and ultimately leads to the occurrence and development of ovarian cancer.^50^ In addition, Kumar et al.^40^ found that AKAP4 (CT99) knockout significantly inhibited the proliferation and viability of ovarian cancer cells, induced cell cycle arrest, and increased the production of reactive oxygen species, DNA damage, and apoptosis in tumor cells, which inhibited tumor growth in animal experiments. Likewise, the ablation of SPAG9 results in reduced cellular proliferation, cell cycle arrest in S phase, and increased apoptosis.^76^ Paclitaxel‐induced apoptosis is enhanced when the expression of HORMAD1, also known as CT46, is disrupted by ovarian cancer cells.^48^ Another study indicated that overexpression of PIWIL2 acts as an oncogene in ovarian cancer by inhibition of apoptosis and promotion of proliferation.^70^

### 5.2 CTA and tumor migration and invasion

Sp17 is called SPA17 or CT22 and plays an important role in the cell transformation, intercellular adhesion, and cell migration of lymphocytes and hematopoietic cells by interacting with extracellular heparin sulfate or binding to another CTA gene, AKAP3 (CT82). The overexpression of Sp17 significantly enhanced the migration ability of ovarian cancer cells HO8910 but decreased the chemotherapy sensitivity of ovarian cancer cells to carboplatin and cisplatin.^94^ Shahzad et al.^48^ interfered HORMAD1 expression by using siRNA, and the results showed that the migration and invasion ability and angiogenesis of ovarian cancer cells were inhibited, accompanying decrease in VEGF and NF‐KB. Other investigators have also observed that Hiwi (PIWIL1) was universally upregulated in ovarian cancer and associated with tumor angiogenesis.^66^ Similarly, TEX19, SPAG9, HSP70‐2, and AKAP4 can promote the invasion and metastasis of ovarian cancer, and the downregulation of these genes can significantly inhibit the migration and invasion ability of ovarian cancer cells.^40^, ^50^, ^76^, ^85^ EMT plays a key role in the invasion and metastasis of ovarian cancer. SSX expression affects EMT in tumor cells, and the downregulation of SSX expression can inhibit the promoting effect of vimentin and MMP2 proteins on tumor cell invasion.^81^

### 5.3 CTA and drug resistance

Platinum combined with paclitaxel chemotherapy is the most important adjuvant therapy in the treatment of ovarian cancer, and drug resistance is the main cause of chemotherapy failure in ovarian cancer. Tumor chemoresistance is associated with increased CTA gene expression. MAGE family genes are highly expressed in some ovarian cancer drug‐resistant cell lines, and the transfection of MAGE‐A2 and MAGE‐A6 into sensitive cell lines can promote cell proliferation and induce their resistance to paclitaxel and adriamycin.^46^ The overexpression of MAGE‐A3 is associated with doxorubicin resistance, and the expression level of MAGE‐C1 is linked to platinum sensitivity in patients with ovarian cancer.^95^ CT45 binds to protein phosphatase 4 and modulates its activity. High expression of CT45 results in DNA damage and increases sensitivity to platinum. After CT45 expression was enforced in ovarian cancer), DNA damage repair gene FANCD2 increased in the S and G2/M phases, indicating increased DNA damage and replication pressure.^96^, ^97^ Coscia et al.^45^ showed that CT45 regulated the sensitivity of ovarian cancer to chemotherapy by affecting the DNA damage repair pathway and may improve the response of patients to immunotargeted therapy; they also found that CT45 is an inherent chemotherapeutic sensitivity enhancer of cells and a nonmutational tumor antigen recognized by cytotoxic T cells. More recently, a cross‐omics analysis revealed that CT45 protein localization closely matched the DNA repair factors 53BP1 and BRCA1. Therefore, the effect of CT45 on the chemotherapy sensitivity of ovarian cancer is closely related to its important role in DNA damage response.^98^ PIWIL2 is a member of the CT80 family, also known as CT80.2, a reproductive stem cell gene. PIWIL2 is considered a protective agent of DNA damage and carcinogenesis, and its expression is associated with cell proliferation and apoptosis inhibition. PIWIL2 overexpression promotes platinum resistance in ovarian cancer cells by enhancing cisplatin‐induced DNA damage repair.^99^, ^100^ Whitehurst et al.^101^ found that OY‐TES‐1 can bind to the mitotic spindle protein NuMA, affecting the assembly of mitotic spindle and promoting the proliferation of tumor cells. The inhibition of OY‐TES‐1 expression can increase the sensitivity of ovarian cancer cells to paclitaxel.^102^ Additional studies have shown that SP17 is associated with chemotherapy resistance of ovarian clear cell carcinoma, and the inhibition of SP17 can enhance the sensitivity of clear cell carcinoma ES‐2 to paclitaxel.^77^, ^94^ In vitro experiments have shown that SPAG9 knockout is associated with ovarian cancer resistance to paclitaxel‐induced cell death.^76^ Duan et al.^83^ first identified highly expressed TRAG‐3 in paclitaxel‐resistant ovarian cancer cell lines; they further confirmed that TRAG‐3 is a CTA. Clinical studies have demonstrated that TRAG‐3 expression in ovarian cancer is closely associated with paclitaxel resistance in patients.^84^

## 6 APPLICATION PROSPECT OF CTA IN OVARIAN CANCER

### 6.1 Biomarkers for tumor diagnosis and prognosis assessment

CTA is highly expressed in ovarian cancer and can partly induce a humoral immune response, which is closely related to clinical stage, histological grade, pathological type, and lymph node metastasis. In addition, CTA shows different expression patterns in specific pathological subtypes or different stages of ovarian cancer, which plays an important role in distinguishing different types and grades of ovarian cancer. Thus, CTA has unique value as a biomarker for screening diagnosis and prognosis assessment of ovarian cancer. For example, NY‐ESO‐1 is associated with an aggressive phenotype of ovarian cancer, which is more common in serous and high‐grade ovarian cancer, and patients with ovarian cancer whose tumors express NY‐ESO‐1 or whose sera contain NY‐ESO‐1 antibody have a worse prognosis. The expression of NY‐ESO‐1 can reflect the degree of tumor infiltration lymphocytes, which is closely related to its strong immunogenicity.^60^ In a retrospective clinical study, the researchers analyzed the prognostic significance of the MAGE‐A family in epithelial ovarian cancer. The results showed that MAGE‐A expression is correlated with pathological type, FIGO stage, and preoperative serum CA125 level. The overall survival of patients with ovarian cancer and MAGE‐A‐positive expression was significantly reduced.^103^ By detecting the expression levels of BAGE, MAGE‐A1, MAGE‐A3, and GAGE1/‐2 in the ascites of patients with ovarian cancer, Hofmann et al.^42^ found that the sensitivity of the combined diagnosis of the four markers was 94% compared with cell morphology alone. In addition, a panel of CTAs (SP17, GAGE, and XAGE‐1D) was inversely associated with progression‐free survival in patients with ovarian cancer.^43^, ^77^, ^86^ PIWI proteins are upregulated in stage III epithelial ovarian cancer and associated with lymph node metastasis.^67^ CT45 and AKAP3 are often highly expressed in advanced and high‐grade ovarian cancer and affect the overall survival and disease‐free survival of patients as independent prognostic factors of epithelial ovarian cancer.^21^, ^38^, ^39^ Notably, a study has analyzed 21 ovarian cancer‐associated CTAs and found that GAGE2, CT45, CCT4, and PRAME cancer/testis antigens were revealed to be correlated with the prognosis for ovarian cancer patients.^104^

### 6.2 Tumor immunotherapy

Immunotherapy, a treatment modality aimed at augmenting the body's natural immune responses to eliminate cancerous cells, stands as a notable advancement in the field of cancer treatment.^105^ Immunotherapy for many solid tumors has been introduced into clinical practice and made remarkable progress. The immune system plays a role in the progression of ovarian cancer, and CTA is a potential target for tumor immunotherapy because of its special expression pattern and immunogenicity. Given that ovarian cancer is one of the cancers with high CTA expression, many patients may benefit from immunotherapy with CTA even after first‐ and second‐line therapy fails.^106^ CTA may apply the following immunotherapeutic strategies:

#### 6.2.1 CTA‐based vaccines

Cancer vaccines are active immunotherapies that either deliver antigens to antigen‐presenting cells (APCs) or directly inject activated APCs into patients, triggering the immune system to initiate a specific antitumor response. The therapeutic effect of a tumor vaccine is related to the immunogenicity of a selected antigen. At present, MAGE, NY‐ESO‐1, and GAGE are the most widely used CTAs in peptide vaccine and dendritic cell (DC) vaccine for ovarian cancer. The clinical trial of the NY‐ESO‐1 vaccine is already underway in patients with ovarian cancer.^88^ An NY‐ESO‐1 peptide ESO_157–170_ can be recognized by HLA‐DP4‐restricted CD4 T cells and HLA‐A2‐ and A24‐restricted CD8 T cells to stimulate Th1 and Th2 CD4^+^ T cell responses in patients with epithelial ovarian cancer. A phase I clinical trial of epithelial ovarian cancer used the NY‐ESO‐1 peptide ESO_157–170_ vaccine. The study showed that the repeated vaccination of patients with remission ovarian cancer can cause a comprehensive immune response of body fluid and T cells.^90^ Another study on patients with high‐risk epithelial ovarian cancer who received NY‐ESO‐1b peptide vaccine after clinical remission through surgery combined with chemotherapy showed that the vaccine can induce specific humoral and T‐cell immune responses in patients who are NY‐ESO‐1 positive or negative.^62^ The most successful example of NY‐ESO‐1‐based immunotherapy was reported by a phase II trial in 22 patients with epithelial ovarian cancer at advanced stages who were at high risk of recurrence or progression; the researchers vaccinated the patients with recombinant vaccinia‐NY‐ESO‐1 (rV‐NY‐ESO‐1) and then administered booster vaccinations with recombinant fowlpox‐NY‐ESO‐1 (rF‐NY‐ESO‐1); after the use of NY‐ESO‐1 vaccine, the median overall survival of immune‐activated patients increased to 48 months, whereas that of patients without immune activation was only 15 months.^63^ The TAG family exhibits broad expression across various malignancies and generates HLA‐A2‐restricted epitopes capable of eliciting a peptide‐specific cytotoxic T lymphocyte response.^82^ In recent years, scholars have developed a nanovaccine composed of exosomes and tumor neoantigens for individualized tumor immunotherapy. Exosomes sourced from autologous serum have been shown to augment the immunogenicity of tumor neoantigens, eliciting a specific antitumor immune response and consequently impeding tumor progression.^107^, ^108^ Since exosomes isolated from ovarian cancer patients' plasma carried MAGE3/6 protein, patients could possibly benefit from the novel therapeutic strategy.^54^

Similarly, Batchu's preclinical study showed that dendritic cells (DCs) transfected with rAAV‐6 capsid mutant vector and cocultured with autologous T lymphocytes and MAGE‐A3‐expressing DCs can induce Cytotoxic T Lymphocyte (CTL) with IFN‐γ‐secreting ability. The CTLs effectively kill MAGE‐A3 positive epithelial ovarian cancer cells.^56^ This DC immunotherapy, either alone or in combination with other immunoenhancement regimens, is of great value to the clinical treatment of ovarian cancer. In addition, two studies demonstrated the tumor inhibition of Sp17 vaccine in a mouse ovarian cancer model. Chiriva‐Internati et al.^109^ immunized mice with the Sp17 protein and found that Sp17 vaccine with CpG deoxynucleotides (CpG ODN) as adjuvant can overcome immune tolerance, inhibit tumor growth, and prolong survival in ovarian cancer mice. The nanoparticle‐based peptide vaccine hSp_17 111–142_ designed by Xiang et al.^78^ can induce Th1/Th2 reaction and stimulate B cells to produce IgG1 and IgG2a. These results still need to be further verified by clinical trials, and we hope that this vaccine can be applied to ovarian cancer in the future. Given that the heterogeneous expression of CTA is common in tumors, the clinical efficacy of vaccines targeting a single antigen will be greatly reduced. Thus, vaccines targeting multiple TAAs must be developed. To date, relevant clinical trials have not yet yielded results.

#### 6.2.2 TCR‐T cell therapy based on CTA

T cells play a key role in cell‐mediated immunity. Adoptive T‐cell immunotherapy is a promising approach to cancer therapy, especially engineered T‐cell receptor (TCR) or chimeric antigen receptor (CAR) gene‐engineered effector T cells. This emerging anticancer immunotherapy has been recognized for its potential benefits for patients with compromised immune systems, as it effectively boosts host immunity.^110^ T cell receptor (TCR) T cell therapy has achieved substantial advances in the treatment of malignant tumors. Cancer immunotherapy using TCR‐engineered T cells targeting TAAs expressed by cancer cells is a promising strategy, especially for solid tumors. According to previous studies, the TAA‐TCR‐T therapy shows considerable potential for treating malignant cancers, such as melanoma, sarcoma, and mesothelioma. The most common TCR‐T cell therapy is TCR‐T cell therapy targeting CTA. NY‐ESO‐1 is currently the most effective target for TCR‐T therapy and is highly expressed at varying frequencies in multiple malignancies, including ovarian cancer.^111^ Many clinical studies have focused on the application of TCR‐T‐targeted CT‐X therapy and achieved satisfactory results. For example, after TCR‐T targeting NY‐ESO‐1 therapy, clinical responses were observed in 66.7% of patients with synovial cell sarcoma and 45.5% of patients with melanoma; two patients with melanoma achieved complete remission, and one patient with synovial cell sarcoma achieved partial remission.^88^ A phase I/II clinical trial of TCR‐T targeting NY‐ESO‐1 in ovarian cancer demonstrated its safety and efficacy.^64^ Rosenberg et al.^57^, ^112^ used TCR‐T targeting MAGE‐A3 to treat a variety of advanced metastatic solid tumors and achieved positive clinical efficacy; they confirmed the efficacy of MHCII‐TCR CD4 T cell therapy targeting MAGE‐A3 and expanded the therapeutic range of TCR‐T for metastatic tumors. Amerongen^72^ and colleagues found that the PRAME and CTCFL TCR‐T cells demonstrated potent and specific antitumor reactivity both in vitro and in vivo, the identified PRAME and CTCFL TCRs are promising candidates for the treatment of patients with ovarian cancer. Novel TCR optimization strategies for multiple tumor antigens have been proposed. Common antigen groups include multiple CTAs (such as NY‐ESO‐1, MAGE‐A1/A4, PRAME, and SSX2). This multiantigen combined TCR‐T cell treatment strategy not only improves antitumor response but also reduces antigen escape and prevents tumor recurrence.

#### 6.2.3 CTA vaccine combined with epigenetic drugs

The expression of immunogenic tumor antigen is one of the key factors affecting the effect of tumor immunotherapy. In tumors, the heterogeneous expression of these tumor antigens is highly frequent and leads to the immune escape of tumor cells with negative or limited expression of antigens; this condition is a barrier to vaccine efficacy and is considered the main reason for the failure of tumor immunotherapy. The ability to induce high levels of CTA expression in all tumor cells would overcome these limitations. Given that CTA expression in tumor tissues is mostly regulated by epigenetic mechanisms (DNA methylation and histone modification), the use of epigenetic drugs to activate CTA, improve its expression rate, and weaken the expression heterogeneity of CTA in tumors will help improve the immunogenicity of tumor cells and enhance the efficacy of CTA targeted therapy. DAC is a DNA methyltransferase inhibitor that inhibits DNA methylation. A series of preclinical studies have shown that using DAC to inhibit the methylation of CpG island in the promoter region of CTA gene upregulates the CTA of tumor cells and enhances the antitumor ability of T cells, laying a foundation for a novel therapy using epigenetic modulators combined with CTA antitumor vaccine. Given that antigen loss is a major escape mechanism during immunotherapy, expanding the repertoire of antigens targeted by immunotherapy approaches seems promising. Adair et al.^113^ demonstrated that the treatment of ovarian cancer cells with DAC can lead to a large increase in the expression of multiple CTA genes, a modest increase in the expression of class I MHC proteins, and enhance the recognition of the treated cells by antigen‐specific CD8^+^ T cells. By conducting a phase I clinical study, Odunsi et al.^65^ found that the use of DAC‐enhanced NY‐ESO‐1 vaccine to treat recurrent ovarian cancer can induce NY‐ESO‐1‐specific CD4^+^ and CD8^+^ T cell responses for up to 12 months. The characteristics of NY‐ESO‐1‐specific CD4^+^ and CD8^+^ TCR were analyzed by vaccine‐induced T cells. Similarly, the CTCFL TCR‐T cells efficiently recognized ovarian cancer cell lines treated with DAC, implying that pretreatment with DAC may increase the reactivity of transferred TCR‐T cells in patients.^72^ Epigenetic regulation not only affects tumor cells but also affects the immune cells of the body. HDAC inhibitors have immune stimulatory effects, and the host immune system also plays an important role in mediating the sustained antitumor response of HDAC inhibitors.^114^ In conclusion, epigenetic drugs combined with immunotherapy have great research potential.

CTA is an ideal target for tumor immunotherapy. Current basic research and clinical trials mainly focus on CTA in antitumor vaccine and adoptive T cell therapy, both of which have advantages and disadvantages. The development trend of CTA‐related immunotherapy is a comprehensive treatment strategy combined with surgery and chemoradiotherapy. With the continuous progress in research, CTA will play a key role in anti‐ovarian cancer immunotherapy.

## 7 SUMMARY AND OUTLOOK

In ovarian cancer, CTA is expressed specifically, which is closely related to clinical prognosis and can affect the occurrence and development of ovarian cancer through numerous aspects, such as cell cycle, apoptosis, migration, and invasion; DNA damage repair; and drug resistance. In addition, CTA has strong immunogenicity and can induce effective humoral and cellular immune responses, suggesting that CTA can be used as a biomarker for the diagnosis and prognosis evaluation of ovarian cancer and is an ideal target for antitumor immunotherapy. Studying the expression regulation and immune process of CTA in ovarian cancer is of great clinical significance. Research into the expression, regulation mechanism, and function of CTA genes in ovarian cancer is still in the primary stage, and few clinical trials of CTA‐based tumor vaccines and adoptive T cell therapies have been conducted, and most of them are still at the level of cell and animal experiments. To comprehensively understand the mechanism and immune process of CTA in ovarian cancer, more clinical trials and research and development of combination therapies targeting CTA are needed. These efforts will contribute to the development of CTA targeted immunotherapy for ovarian cancer and provide novel approaches for the personalized treatment of ovarian cancer. Developments in research into CTA as a targeted treatment strategy for ovarian cancer may offer considerable benefits to patients with ovarian cancer.

## AUTHOR CONTRIBUTIONS

**Lina Lin**: Resources; writing—original draft; writing—review and editing. **Xiaoqiong Zou**: Writing—review and editing. **Weixia Nong**: Funding acquisition. **Yingying Ge**: Resources. **Feng Li**: Resources. **Bin Luo**: Project administration. **Qingmei Zhang**: Project administration. **Xiaoxun Xie**: Project administration; writing—review and editing.

## References

[1] Arnaoutoglou C , Dampala K , Anthoulakis C , et al. Epithelial ovarian cancer: a five year review. Medicina. 2023;59:1183. 37511995 10.3390/medicina59071183 PMC10384230

[2] Sung H , Ferlay J , Siegel RL , et al. Global cancer statistics 2020: GLOBOCAN estimates of incidence and mortality worldwide for 36 cancers in 185 countries. CA Cancer J Clin. 2021;71:209‐249. 33538338 10.3322/caac.21660

[3] Torre LA , Trabert B , DeSantis CE , et al. Ovarian cancer statistics, 2018. CA Cancer J Clin. 2018;68(4):284‐296. 29809280 10.3322/caac.21456 PMC6621554

[4] Stewart C , Ralyea C , Lockwood S . Ovarian cancer: an integrated review. Semin Oncol Nurs. 2019;35(2):151‐156. 30867104 10.1016/j.soncn.2019.02.001

[5] Gil‐Martin M , Pardo B , Barretina‐Ginesta MP . Rare ovarian tumours. Other treatments for ovarian cancer. EJC Suppl. 2020;15:96‐103. 33240448 10.1016/j.ejcsup.2019.11.002 PMC7573466

[6] Morand S , Devanaboyina M , Staats H , Stanbery L , Nemunaitis J . Ovarian cancer immunotherapy and personalized medicine. Int J Mol Sci. 2021;22(12):6532. 34207103 10.3390/ijms22126532 PMC8234871

[7] Costa FF , Le Blanc K , Brodin B . Concise review: cancer/testis antigens, stem cells, and cancer. Stem Cells. 2007;25(3):707‐711. 17138959 10.1634/stemcells.2006-0469

[8] Xie K , Fu C , Wang S , et al. Cancer‐testis antigens in ovarian cancer: implication for biomarkers and therapeutic targets. J Ovarian Res. 2019;12(1):1. 30609934 10.1186/s13048-018-0475-z PMC6318940

[9] Zendman AJW , Ruiter DJ , Van Muijen GNP . Cancer/testis‐associated genes: identification, expression profile, and putative function. J Cell Physiol. 2003;194(3):272‐288. 12548548 10.1002/jcp.10215

[10] Kim R , Kulkarni P , Hannenhalli S . Derepression of cancer/testis antigens in cancer is associated with distinct patterns of DNA hypomethylation. BMC Cancer. 2013;13:144. 23522060 10.1186/1471-2407-13-144 PMC3618251

[11] De Smet C , Lurquin C , Lethé B , Martelange V , Boon T . DNA methylation is the primary silencing mechanism for a set of germ line‐ and tumor‐specific genes with a CpG‐Rich promoter. Mol Cell Biol. 1999;19(11):7327‐7335. 10523621 10.1128/mcb.19.11.7327 PMC84726

[12] Menendez L , Walker D , Matyunina LV , et al. Identification of candidate methylation‐responsive genes in ovarian cancer. Mol Cancer. 2007;6:10. 17254359 10.1186/1476-4598-6-10 PMC1803786

[13] De Smet C , De Backer O , Faraoni I , Lurquin C , Brasseur F , Boon T . The activation of human gene MAGE‐1 in tumor cells is correlated with genome‐wide demethylation. Proc Natl Acad Sci USA. 1996;93(14):7149‐7153. 8692960 10.1073/pnas.93.14.7149 PMC38951

[14] Yegnasubramanian S , Haffner MC , Zhang Y , et al. DNA hypomethylation arises later in prostate cancer progression than CpG island hypermethylation and contributes to metastatic tumor heterogeneity. Cancer Res. 2008;68(21):8954‐8967. 18974140 10.1158/0008-5472.CAN-07-6088 PMC2577392

[15] Roman‐Gomez J , Jimenez‐Velasco A , Agirre X , et al. Epigenetic regulation of human cancer/testis antigen gene, HAGE, in chronic myeloid leukemia. Haematologica. 2007;92(2):153‐162. 17296563 10.3324/haematol.10782

[16] Cho B , Lee H , Jeong S , et al. Promoter hypomethylation of a novel cancer/testis antigen gene CAGE is correlated with its aberrant expression and is seen in premalignant stage of gastric carcinoma. Biochem Biophys Res Commun. 2003;307(1):52‐63. 12849980 10.1016/s0006-291x(03)01121-5

[17] Woloszynska‐Read A , James SR , Song C , Jin B , Odunsi K , Karpf AR . BORIS/CTCFL expression is insufficient for cancer‐germline antigen gene expression and DNA hypomethylation in ovarian cell lines. Cancer Immun. 2010;10(10):6. 20649179 PMC2916237

[18] Link PA , Zhang W , Odunsi K , Karpf AR . BORIS/CTCFL mRNA isoform expression and epigenetic regulation in epithelial ovarian cancer. Cancer Immun. 2013;13(6):6. 23390377 PMC3559194

[19] Woloszynska‐Read A , Mhawech‐Fauceglia P , Yu J , Odunsi K , Karpf AR . Intertumor and intratumor NY‐ESO‐1 expression heterogeneity is associated with promoter‐specific and global DNA methylation status in ovarian cancer. Clin Cancer Res. 2008;14(11):3283‐3290. 18519754 10.1158/1078-0432.CCR-07-5279 PMC2835568

[20] Woloszynska‐Read A , Zhang W , Yu J , et al. Coordinated cancer germline antigen promoter and global DNA hypomethylation in ovarian cancer: association with the BORIS/CTCF expression ratio and advanced stage. Clin Cancer Res. 2011;17(8):2170‐2180. 21296871 10.1158/1078-0432.CCR-10-2315 PMC3079045

[21] Zhang W , Barger CJ , Link PA , et al. DNA hypomethylation‐mediated activation of cancer/testis antigen 45 (CT45) genes is associated with disease progression and reduced survival in epithelial ovarian cancer. Epigenetics. 2015;10(8):736‐748. 26098711 10.1080/15592294.2015.1062206 PMC4622579

[22] Zhang W , Barger CJ , Eng KH , et al. PRAME expression and promoter hypomethylation in epithelial ovarian cancer. Oncotarget. 2016;7(29):45352‐45369. 27322684 10.18632/oncotarget.9977 PMC5216727

[23] Rao M , Chinnasamy N , Hong JA , et al. Inhibition of histone lysine methylation enhances cancer‐testis antigen expression in lung cancer cells: implications for adoptive immunotherapy of cancer. Cancer Res. 2011;71(12):4192‐4204. 21546573 10.1158/0008-5472.CAN-10-2442 PMC3116976

[24] Link PA , Gangisetty O , James SR , et al. Distinct roles for histone methyltransferases G9a and GLP in cancer germ‐line antigen gene regulation in human cancer cells and murine embryonic stem cells. Mol Cancer Res. 2009;7(6):851‐862. 19531572 10.1158/1541-7786.MCR-08-0497 PMC2836864

[25] Steele N , Finn P , Brown R , Plumb JA . Combined inhibition of DNA methylation and histone acetylation enhances gene re‐expression and drug sensitivity in vivo. Br J Cancer. 2009;100(5):758‐763. 19259094 10.1038/sj.bjc.6604932 PMC2653770

[26] Devor EJ , Gonzalez‐Bosquet J , Warrier A , et al. p53 mutation status is a primary determinant of placenta‐specific protein 1 expression in serous ovarian cancers. Int J Oncol. 2017;50(5):1721‐1728. 28339050 10.3892/ijo.2017.3931 PMC5403493

[27] Renaud S , Pugacheva EM , Delgado MD , et al. Expression of the CTCF‐paralogous cancer‐testis gene, brother of the regulator of imprinted sites (BORIS), is regulated by three alternative promoters modulated by CpG methylation and by CTCF and p53 transcription factors. Nucleic Acids Res. 2007;35(21):7372‐7388. 17962299 10.1093/nar/gkm896 PMC2175345

[28] Pageau GJ , Hall LL , Ganesan S , Livingston DM , Lawrence JB . The disappearing Barr body in breast and ovarian cancers. Nat Rev Cancer. 2007;7(8):628‐633. 17611545 10.1038/nrc2172

[29] Cheng PC , Gosewehr JA , Kim TM , et al. Potential role of the inactivated X chromosome in ovarian epithelial tumor development. J Natl Cancer Inst. 1996;88(8):510‐518. 8606379 10.1093/jnci/88.8.510

[30] Kang J , Lee HJ , Kim J , Lee JJ , Maeng L . Dysregulation of X chromosome inactivation in high grade ovarian serous adenocarcinoma. PLoS One. 2015;10(3):e0118927. 25742136 10.1371/journal.pone.0118927 PMC4351149

[31] Wang Z , Zhang J , Zhang Y , Lim SH . SPAN‐Xb expression in myeloma cells is dependent on promoter hypomethylation and can be upregulated pharmacologically. Int J Cancer. 2006;118(6):1436‐1444. 16187275 10.1002/ijc.21499

[32] Yang B , Wu J , Maddodi N , Ma Y , Setaluri V , Jack Longley B . Epigenetic control of MAGE gene expression by the KIT tyrosine kinase. J Invest Dermatol. 2007;127(9):2123‐2128. 17495964 10.1038/sj.jid.5700836

[33] Tammela J , Uenaka A , Ono T , et al. OY‐TES‐1 expression and serum immunoreactivity in epithelial ovarian cancer. Int J Oncol. 2006;29(4):903‐910. 16964386

[34] Fan R , Huang W , Luo B , Zhang QM , Xiao SW , Xie XX . Cancer testis antigen OY‐TES‐1: analysis of protein expression in ovarian cancer with tissue microarrays. Eur J Gynaecol Oncol. 2015;36(3):298‐303. 26189257

[35] Garcia‐Soto AE , Schreiber T , Strbo N , et al. Cancer‐testis antigen expression is shared between epithelial ovarian cancer tumors. Gynecol Oncol. 2017;145(3):413‐419. 28392126 10.1016/j.ygyno.2017.03.512

[36] Luo B , Yun X , Li J , et al. Cancer‐testis antigen OY‐TES‐1 expression and immunogenicity in hepatocellular carcinoma. Curr Med Sci. 2020;40(4):719‐728. 32862383 10.1007/s11596-020-2241-x

[37] Okumura H , Noguchi Y , Uenaka A , et al. Identification of an HLA‐A24‐restricted OY‐TES‐1 epitope recognized by cytotoxic T‐cells. Microbiol Immunol. 2005;49(11):1009‐1016. 16301813 10.1111/j.1348-0421.2005.tb03688.x

[38] Sharma S , Qian F , Keitz B , et al. A‐kinase anchoring protein 3 messenger RNA expression correlates with poor prognosis in epithelial ovarian cancer. Gynecol Oncol. 2005;99(1):183‐188. 16005946 10.1016/j.ygyno.2005.06.006

[39] Hasegawa K , Ono T , Matsushita H , et al. A‐kinase anchoring protein 3 messenger RNA expression in ovarian cancer and its implication on prognosis. Int J Cancer. 2004;108(1):86‐90. 14618620 10.1002/ijc.11565

[40] Agarwal S , Saini S , Parashar D , et al. The novel cancer‐testis antigen A‐kinase anchor protein 4 (AKAP4) is a potential target for immunotherapy of ovarian serous carcinoma. Oncoimmunology. 2013;2(5):e24270. 23762804 10.4161/onci.24270 PMC3667910

[41] Zhang S , Zhou X , Yu H , Yu Y . Expression of tumor‐specific antigen MAGE, GAGE and BAGE in ovarian cancer tissues and cell lines. BMC Cancer. 2010;10:163. 20423514 10.1186/1471-2407-10-163 PMC2868811

[42] Hofmann M , Ruschenburg I . mRNA detection of tumor‐rejection genes BAGE, GAGE, and MAGE in peritoneal fluid from patients with ovarian carcinoma as a potential diagnostic tool. Cancer. 2002;96(3):187‐193. 12115308 10.1002/cncr.10622

[43] Gillespie A , Rodgers S , Wilson A , et al. MAGE, BAGE and GAGE: tumour antigen expression in benign and malignant ovarian tissue. Br J Cancer. 1998;78(6):816‐821. 9743307 10.1038/bjc.1998.585 PMC2062964

[44] Chen YT , Hsu M , Lee P , et al. Cancer/testis antigen CT45: analysis of mRNA and protein expression in human cancer. Int J Cancer. 2009;124(12):2893‐2898. 19296537 10.1002/ijc.24296

[45] Coscia F , Lengyel E , Duraiswamy J , et al. Multi‐level proteomics identifies CT45 as a chemosensitivity mediator and immunotherapy target in ovarian. Cell. 2018;175(1):159‐170. 30241606 10.1016/j.cell.2018.08.065 PMC6827878

[46] Duan Z , Duan Y , Lamendola DE , et al. Overexpression of MAGE/GAGE genes in paclitaxel/doxorubicin‐resistant human cancer cell lines. Clin Cancer Res. 2003;9(7):2778‐2785. 12855658

[47] Türeci Ö , Sahin U , Koslowski M , et al. A novel tumour associated leucine zipper protein targeting to sites of gene transcription and splicing. Oncogene. 2002;21(24):3879‐3888. 12032826 10.1038/sj.onc.1205481

[48] Shahzad MMK , Shin YH , Matsuo K , et al. Biological significance of HORMA domain containing protein 1 (HORMAD1) in epithelial ovarian carcinoma. Cancer Lett. 2013;330(2):123‐129. 22776561 10.1016/j.canlet.2012.07.001 PMC3498611

[49] Zhu D , Dix DJ , Eddy EM . HSP70‐2 is required for CDC2 kinase activity in meiosis I of mouse spermatocytes. Development. 1997;124(15):3007‐3014. 9247342 10.1242/dev.124.15.3007

[50] Gupta N , Jagadish N , Surolia A , Suri A . Heat shock protein 70‐2 (HSP70‐2) a novel cancer testis antigen that promotes growth of ovarian cancer. Am J Cancer Res. 2017;7(6):1252‐1269. 28670489 PMC5489776

[51] Odunsi K , Jungbluth AA , Stockert E , et al. NY‐ESO‐1 and LAGE‐1 cancer‐testis antigens are potential targets for immunotherapy in epithelial ovarian cancer. Cancer Res. 2003;63(18):6076‐6083. 14522938

[52] Yamada A , Kataoka A , Shichijo S , et al. Expression of MAGE‐1, MAGE‐2, MAGE‐3/‐6 and MAGE‐4a/‐4b genes in ovarian tumors. Int J Cancer. 1995;64(6):388‐393. 8550240 10.1002/ijc.2910640607

[53] Daudi S , Eng KH , Mhawech‐Fauceglia P , et al. Expression and immune responses to MAGE antigens predict survival in epithelial ovarian cancer. PLoS One. 2014;9(8):e104099. 25101620 10.1371/journal.pone.0104099 PMC4125181

[54] Szajnik M , Derbis M , Lach M , et al. Exosomes in plasma of patients with ovarian carcinoma: potential biomarkers of tumor progression and response to therapy. Gynecol Obstetr. 2013;4:3. 10.4172/2161-0932.S4-003 PMC3899646 24466501

[55] Esfandiary A , Ghafouri‐Fard S . MAGE‐A3: an immunogenic target used in clinical practice. Immunotherapy. 2015;7(6):683‐704. 26100270 10.2217/imt.15.29

[56] Batchu RB , Gruzdyn OV , Moreno‐Bost AM , et al. Efficient lysis of epithelial ovarian cancer cells by MAGE‐A3‐induced cytotoxic T lymphocytes using rAAV‐6 capsid mutant vector. Vaccine. 2014;32(8):938‐943. 24406390 10.1016/j.vaccine.2013.12.049

[57] Lu YC , Parker LL , Lu T , et al. Treatment of patients with metastatic cancer using a major histocompatibility complex class II‐restricted T‐cell receptor targeting the cancer germline antigen MAGE‐A3. J Clin Oncol. 2017;35(29):3322‐3329. 28809608 10.1200/JCO.2017.74.5463 PMC5652397

[58] Kawagoe H , Yamada A , Matsumoto H , et al. Serum MAGE‐4 protein in ovarian cancer patients. Gynecol Oncol. 2000;76(3):336‐339. 10684707 10.1006/gyno.1999.5701

[59] Xu Y , Wang C , Zhang Y , Jia L , Huang J . Overexpression of MAGE‐A9 is predictive of poor prognosis in epithelial ovarian cancer. Sci Rep. 2015;5:12104. 26175056 10.1038/srep12104 PMC4502509

[60] Szender JB , Papanicolau‐Sengos A , Eng KH , et al. NY‐ESO‐1 expression predicts an aggressive phenotype of ovarian cancer. Gynecol Oncol. 2017;145(3):420‐425. 28392127 10.1016/j.ygyno.2017.03.509 PMC5497581

[61] Odunsi K. Kunle , Qian F , Matsuzaki J , et al. Vaccination with an NY‐ESO‐1 peptide of HLA class I/II specificities induces integrated humoral and T cell responses in ovarian cancer. Proc Natl Acad Sci USA. 2007;104(31):12837‐12842. 17652518 10.1073/pnas.0703342104 PMC1937553

[62] Diefenbach CSM , Gnjatic S , Sabbatini P , et al. Safety and immunogenicity study of NY‐ESO‐1b peptide and montanide ISA‐51 vaccination of patients with epithelial ovarian cancer in high‐risk first remission. Clin Cancer Res. 2008;14(9):2740‐2748. 18451240 10.1158/1078-0432.CCR-07-4619

[63] Odunsi K , Matsuzaki J , Karbach J , et al. Efficacy of vaccination with recombinant vaccinia and fowlpox vectors expressing NY‐ESO‐1 antigen in ovarian cancer and melanoma patients. Proc Natl Acad Sci USA. 2012;109(15):5797‐5802. 22454499 10.1073/pnas.1117208109 PMC3326498

[64] Robbins PF , Morgan RA , Feldman SA , et al. Tumor regression in patients with metastatic synovial cell sarcoma and melanoma using genetically engineered lymphocytes reactive with NY‐ESO‐1. J Clin Oncol. 2011;29(7):917‐924. 21282551 10.1200/JCO.2010.32.2537 PMC3068063

[65] Odunsi K , Matsuzaki J , James SR , et al. Epigenetic potentiation of NY‐ESO‐1 vaccine therapy in human ovarian cancer. Cancer Immunol Res. 2014;2(1):37‐49. 24535937 10.1158/2326-6066.CIR-13-0126 PMC3925074

[66] Li S , Meng L , Zhu C , et al. The universal overexpression of a cancer testis antigen hiwi is associated with cancer angiogenesis. Oncol Rep. 2010;23(4):1063‐1068. 20204292

[67] Chen C , Liu J , Xu G , et al. Overexpression of PIWI proteins in human stage III epithelial ovarian cancer with lymph node metastasis. Cancer Biomarkers. 2013;13(5):315‐321. 24440970 10.3233/CBM-130360 PMC12928306

[68] Lu L , Katsaros D , Risch HA , Canuto EM , Biglia N , Yu H . MicroRNA let‐7a modifies the effect of self‐renewal gene HIWI on patient survival of epithelial ovarian cancer. Mol Carcinog. 2016;55(4):357‐365. 25630839 10.1002/mc.22285

[69] Lim SL , Ricciardelli C , Oehler MK , De Arao Tan IMD , Russell D , Grützner F . Overexpression of piRNA pathway genes in epithelial ovarian cancer. PLoS One. 2014;9(6):e99687. 24932571 10.1371/journal.pone.0099687 PMC4059699

[70] Lee JH , Schütte D , Wulf G , et al. Stem‐cell protein Piwil2 is widely expressed in tumors and inhibits apoptosis through activation of Stat3/Bcl‐XL pathway. Hum Mol Gen. 2006;15(2):201‐211. 16377660 10.1093/hmg/ddi430

[71] Kaczorowski M , Chłopek M , Kruczak A , Ryś J , Lasota J , Miettinen M . PRAME expression in cancer. A systematic immunohistochemical study of >5800 epithelial and nonepithelial tumors. Am J Surg Pathol. 2022;46(11):1467‐1476. 35973038 10.1097/PAS.0000000000001944 PMC9588667

[72] van Amerongen RA , Tuit S , Wouters AK , et al. PRAME and CTCFL‐reactive TCRs for the treatment of ovarian cancer. Front Immunol. 2023;14:1121973. 37026005 10.3389/fimmu.2023.1121973 PMC10070997

[73] Tchabo NE , Mhawech‐Fauceglia P , Caballero OL , et al. Expression and serum immunoreactivity of developmentally restricted differentiation antigens in epithelial ovarian cancer. Cancer Immun. 2009;9:6. 19705800 PMC2935768

[74] Wang X , Baddoo MC , Yin Q . The placental specific gene, PLAC1, is induced by the Epstein‐Barr virus and is expressed in human tumor cells. Virol J. 2014;11:107. 24912876 10.1186/1743-422X-11-107 PMC4072619

[75] Barger CJ , Zhang W , Sharma A , et al. Expression of the POTE gene family in human ovarian cancer. Sci Rep. 2018;8(1):17136. 30459449 10.1038/s41598-018-35567-1 PMC6244393

[76] Jagadish N , Fatima R , Sharma A , et al. Sperm associated antigen 9 (SPAG9) a promising therapeutic target of ovarian carcinoma. Tumor Biol. 2018;40(5):101042831877365. 10.1177/1010428318773652 29745297

[77] Li F , Han Y , Liu Q , Wu B , Huang W , Zeng S . Overexpression of human sperm protein 17 increases migration and decreases the chemosensitivity of human epithelial ovarian cancer cells. BMC Cancer. 2009;9:323. 19744347 10.1186/1471-2407-9-323 PMC2753635

[78] Xiang S , Gao Q , Wilson K , Heyerick A , Plebanski M . A nanoparticle based Sp17 peptide vaccine exposes new immuno‐dominant and species crossreactive B cell epitopes. Vaccines. 2015;3(4):875‐893. 26529027 10.3390/vaccines3040875 PMC4693223

[79] Straughn, Jr. JM , Shaw DR , Guerrero A , et al. Expression of sperm protein 17 (Sp17) in ovarian cancer. Int J Cancer. 2004;108(6):805‐811. 14712480 10.1002/ijc.11617

[80] Valmori D , Qian F , Ayyoub M , et al. Expression of synovial sarcoma X (SSX) antigens in epithelial ovarian cancer and identification of SSX‐4 epitopes recognized by CD4+T cells. Clin Cancer Res. 2006;12(2):398‐404. 16428478 10.1158/1078-0432.CCR-05-1902

[81] Hasegawa K , Koizumi F , Noguchi Y , et al. SSX expression in gynecological cancers and antibody response in patients. Cancer Immun. 2004;4:16. 15603546

[82] Adair SJ , Carr TM , Fink MJ , Slingluff CL , Hogan KT . The TAG family of cancer/testis antigens is widely expressed in a variety of malignancies and gives rise to HLA‐A2‐restricted epitopes. J Immunother. 2008;31(1):7‐17. 18157007 10.1097/CJI.0b013e318159f797

[83] Duan Z , Feller AJ , Toh HC , Makastorsis T , Seiden MV . TRAG‐3, a novel gene, isolated from a taxol‐resistant ovarian carcinoma cell line. Gene. 1999;229(1‐2):75‐81. 10095106 10.1016/s0378-1119(99)00042-6

[84] Materna V , Surowiak P , Kaplenko I , et al. Taxol‐resistance‐associated gene‐3 (TRAG‐3/CSAG2) expression is predictive for clinical outcome in ovarian carcinoma patients. Virchows Arch. 2007;450(2):187‐194. 17216190 10.1007/s00428-006-0346-7

[85] Xu Z , Tang H , Zhang T , et al. TEX19 promotes ovarian carcinoma progression and is a potential target for epitope vaccine immunotherapy. Life Sci. 2020;241:117171. 31843525 10.1016/j.lfs.2019.117171

[86] Jumaa MG , Abdul‐Kareem ramadhan M . Cancer testis antigen XAGE‐1 is a promising marker for the diagnosis and treatment of ovarian cancer. J Med Life. 2021;14(5):710‐715. 35027975 10.25122/jml-2021-0304 PMC8742904

[87] Garg M , Chaurasiya D , Rana R , et al. Sperm‐associated antigen 9, a novel cancer testis antigen, is a potential target for immunotherapy in epithelial ovarian cancer. Clin Cancer Res. 2007;13(5):1421‐1428. 17332284 10.1158/1078-0432.CCR-06-2340

[88] Ali E , Soudeh GF . New York esophageal squamous cell carcinoma‐1 and cancer immunotherapy. Immunotherapy. 2015;7(3):411‐439. 25917631 10.2217/imt.15.3

[89] Ono T , Kurashige T , Harada N , et al. Identification of proacrosin binding protein sp32 precursor as a human cancer/testis antigen. Proc Natl Acad Sci USA. 2001;98(6):3282‐3287. 11248070 10.1073/pnas.041625098 PMC30645

[90] Lin L , Nong W , Luo B , et al. Cancer‐testis antigen ACRBP expression and serum immunoreactivity in ovarian cancer: its association with prognosis. Immun Inflamm Dis. 2021;9(4):1759‐1770. 34528758 10.1002/iid3.534 PMC8589352

[91] Ge YY , Zhang QM , Liu C , et al. Combined treatment with epigenetic agents enhances anti‐tumor activity of T cells by upregulating the ACRBP expression in hepatocellular carcinoma. Am J Transl Res. 2021;13(7):7591‐7609. 34377237 PMC8340224

[92] Wei R , Dean DC , Thanindratarn P , Hornicek FJ , Guo W , Duan Z . Cancer testis antigens in sarcoma: expression, function and immunotherapeutic application. Cancer Lett. 2020;479:54‐60. 31634526 10.1016/j.canlet.2019.10.024

[93] Kulkarni P , Uversky V . Cancer/Testis antigens: “smart” biomarkers for diagnosis and prognosis of prostate and other cancers. Int J Mol Sci. 2017;18(4):740. 28362316 10.3390/ijms18040740 PMC5412325

[94] Nakazato T , Kanuma T , Tamura T , Faried LS , Aoki H , Minegishi T . Sperm protein 17 influences the tissue‐specific malignancy of clear cell adenocarcinoma in human epithelial ovarian cancer. Int J Gynecol Cancer. 2007;17(2):426‐432. 17309563 10.1111/j.1525-1438.2007.00815.x

[95] Bertram J , Palfner K , Hiddemann W , Kneba M . Elevated expression of S100P, CAPL and MAGE 3 in doxorubicin‐resistant cell lines: comparison of mRNA differential display reverse transcription‐polymerase chain reaction and subtractive suppressive hybridization for the analysis of differential gene expre. Anti‐Cancer Drugs. 1998;9(4):311‐318. 9635921 10.1097/00001813-199804000-00004

[96] Hein MY , Hubner NC , Poser I , et al. A human interactome in three quantitative dimensions organized by stoichiometries and abundances. Cell. 2015;163(3):712‐723. 26496610 10.1016/j.cell.2015.09.053

[97] Reed E , Ozols RF , Tarone R , Yuspa SH , Poirier MC . Platinum‐DNA adducts in leukocyte DNA correlate with disease response in ovarian cancer patients receiving platinum‐based chemotherapy. Proc Natl Acad Sci USA. 1987;84(14):5024‐5028. 3110781 10.1073/pnas.84.14.5024 PMC305239

[98] Gupta R , Somyajit K , Narita T , et al. DNA repair network analysis reveals shieldin as a key regulator of NHEJ and PARP inhibitor sensitivity. Cell. 2018;173(4):972‐988.e23. 29656893 10.1016/j.cell.2018.03.050 PMC8108093

[99] Afsharpad M , Nowroozi MR , Mobasheri MB , et al. Cancer‐testis antigens as new candidate diagnostic biomarkers for transitional cell carcinoma of bladder. Pathol Oncol Res. 2019;25(1):191‐199. 29058301 10.1007/s12253-017-0313-4

[100] Wang QE , Han C , Milum K , Wani AA . Stem cell protein Piwil2 modulates chromatin modifications upon cisplatin treatment. Mut Res. 2011;708(1‐2):59‐68. 21310163 10.1016/j.mrfmmm.2011.02.001 PMC3091508

[101] Whitehurst AW , Xie Y , Purinton SC , et al. Tumor antigen acrosin binding protein normalizes mitotic spindle function to promote cancer cell proliferation. Cancer Res. 2010;70(19):7652‐7661. 20876808 10.1158/0008-5472.CAN-10-0840 PMC2948627

[102] Brüning‐Richardson A , Bond J , Alsiary R , et al. NuMA overexpression in epithelial ovarian cancer. PLoS One. 2012;7(6):e38945. 22719996 10.1371/journal.pone.0038945 PMC3375276

[103] Sang M , Lian Y , Zhou X , Shan B . MAGE‐A family: attractive targets for cancer immunotherapy. Vaccine. 2011;29(47):8496‐8500. 21933694 10.1016/j.vaccine.2011.09.014

[104] Vlasenkova R , Konysheva D , Nurgalieva A , Kiyamova R . Characterization of cancer/testis antigens as prognostic markers of ovarian cancer. Diagnostics. 2023;13(19):3092. 37835834 10.3390/diagnostics13193092 PMC10572515

[105] Xie J , Ye F , Deng X , et al. Circular RNA: a promising new star of vaccine. J Transl Int Med. 2023;11(4):372‐381. 38130633 10.2478/jtim-2023-0122 PMC10732498

[106] Yang P , Meng M , Zhou Q . Oncogenic cancer/testis antigens are a hallmarker of cancer and a sensible target for cancer immunotherapy. Biochim Biophys Acta Rev Cancer. 2021;1876(1):188558. 33933558 10.1016/j.bbcan.2021.188558

[107] Xie J , Zheng Z , Tuo L , et al. Recent advances in exosome‐based immunotherapy applied to cancer. Front Immunol. 2023;14:1296857. 38022585 10.3389/fimmu.2023.1296857 PMC10662326

[108] Zhang Y , Zuo B , Yu Z , et al. Complete remission of tumors in mice with neoantigen‐painted exosomes and anti‐PD‐1 therapy. Mol Ther. 2023;31(12):3579‐3593. 37919900 10.1016/j.ymthe.2023.10.021 PMC10727972

[109] Chiriva‐Internati M , Yu Y , Mirandola L , et al. Cancer testis antigen vaccination affords long‐term protection in a murine model of ovarian cancer. PLoS One. 2010;5(5):e10471. 20485677 10.1371/journal.pone.0010471 PMC2868870

[110] Ye F , Dewanjee S , Li Y , et al. Advancements in clinical aspects of targeted therapy and immunotherapy in breast cancer. Mol Cancer. 2023;22(1):105. 37415164 10.1186/s12943-023-01805-y PMC10324146

[111] Fan C , Qu H , Wang X , et al. Cancer/testis antigens: from serology to mRNA cancer vaccine. Sem Cancer Biol. 2021;76:218‐231. 10.1016/j.semcancer.2021.04.016 33910064

[112] Cameron BJ , Gerry AB , Dukes J , et al. Identification of a Titin‐derived HLA‐A1‐presented peptide as a cross‐reactive target for engineered MAGE A3‐directed T cells. Sci Transl Med. 2013;5(197):197ra03. 10.1126/scitranslmed.3006034 PMC6002776 23926201

[113] Adair SJ , Hogan KT . Treatment of ovarian cancer cell lines with 5‐aza‐2′‐deoxycytidine upregulates the expression of cancer‐testis antigens and class I major histocompatibility complex‐encoded molecules. Cancer Immunol Immunother. 2009;58(4):589‐601. 18791715 10.1007/s00262-008-0582-6 PMC11029901

[114] West AC , Mattarollo SR , Shortt J , et al. An intact immune system is required for the anticancer activities of histone deacetylase inhibitors. Cancer Res. 2013;73(24):7265‐7276. 24158093 10.1158/0008-5472.CAN-13-0890

